# Global burden of maternal and congenital syphilis in 2008 and 2012: a health systems modelling study

**DOI:** 10.1016/S2214-109X(16)30135-8

**Published:** 2016-08

**Authors:** N Saman Wijesooriya, Roger W Rochat, Mary L Kamb, Prasad Turlapati, Marleen Temmerman, Nathalie Broutet, Lori M Newman

**Affiliations:** Department of Reproductive Health and Research, World Health Organization, Geneva, Switzerland; Emory University, Rollins School of Public Health, Hubert Department of Global Health, Atlanta, GA, USA; Centers for Disease Control and Prevention, Division of Sexually Transmitted Disease Prevention, Atlanta, GA, USA; Emory University, Rollins School of Public Health, Hubert Department of Global Health, Atlanta, GA, USA; Centers for Disease Control and Prevention, Division of Sexually Transmitted Disease Prevention, Atlanta, GA, USA; Indian Ministry of Health and Family Welfare, National AIDS Control Program, New Delhi, India; Department of Reproductive Health and Research, World Health Organization, Geneva, Switzerland; Department of Reproductive Health and Research, World Health Organization, Geneva, Switzerland; Department of Reproductive Health and Research, World Health Organization, Geneva, Switzerland

## Abstract

**Summary:**

**Background:**

In 2007, WHO launched a global initiative for the elimination of mother-to-child transmission of syphilis (congenital syphilis). An important aspect of the initiative is strengthening surveillance to monitor progress towards elimination. In 2008, using a health systems model with country data inputs, WHO estimated that 1 4 million maternal syphilis infections caused 520 000 adverse pregnancy outcomes. To assess progress, we updated the 2008 estimates and estimated the 2012 global prevalence and cases of maternal and congenital syphilis.

**Methods:**

We used a health systems model approved by the Child Health Epidemiology Reference Group. WHO and UN databases provided inputs on livebirths, antenatal care coverage, and syphilis testing, seropositivity, and treatment in antenatal care. For 2012 estimates, we used data collected between 2009 and 2012. We updated the 2008 estimates using data collected between 2000 and 2008, compared these with 2012 estimates using data collected between 2009 and 2012, and performed subanalyses to validate results.

**Findings:**

In 2012, an estimated 930 000 maternal syphilis infections caused 350 000 adverse pregnancy outcomes including 143 000 early fetal deaths and stillbirths, 62 000 neonatal deaths, 44 000 preterm or low weight births, and 102 000 infected infants worldwide. Nearly 80% of adverse outcomes (274 000) occurred in women who received antenatal care at least once. Comparing the updated 2008 estimates with the 2012 estimates, maternal syphilis decreased by 38% (from 1 488 394 cases in 2008 to 927 936 cases in 2012) and congenital syphilis decreased by 39% (from 576 784 to 350 915). India represented 65% of the decrease. Analysis excluding India still showed an 18% decrease in maternal and congenital cases of syphilis worldwide.

**Interpretation:**

Maternal and congenital syphilis decreased worldwide from 2008 to 2012, which suggests progress towards the elimination of mother-to-child transmission of syphilis. Nonetheless, maternal syphilis caused substantial adverse pregnancy outcomes, even in women receiving antenatal care. Improved access to quality antenatal care, including syphilis testing and treatment, and robust data are all important for achieving the elimination of mother-to-child transmission of syphilis.

**Funding:**

The UNDP-UNFPA-UNICEF-WHO-World Bank Special Programme of Research, Development and Research Training in Human Reproduction in WHO, and the US Centers for Disease Control and Prevention.

## Introduction

Syphilis, a bacterial infection caused by the spirochete *Treponema pallidum,* can be transmitted sexually or from mother to child in utero. Untreated primary or secondary syphilis infections can induce catastrophic fetal outcomes in the second or third trimesters of pregnancy.^1^ Most maternal syphilis infections are latent (asymptomatic), but still result in poor pregnancy outcomes in more than 50% of cases.^2–5^ Syphilis-associated adverse pregnancy outcomes (ie, congenital syphilis) include early fetal death, stillbirth, preterm birth, low birthweight, neonatal death, and congenital infection in infants.

A combination of serological tests measuring infection activity and confirming exposure to *T pallidum* can detect asymptomatic syphilis infections in pregnant women. Maternal syphilis screening and treatment of seropositive women with at least 2.4 million units of benzathine penicillin prevents most adverse pregnancy outcomes if provided sufficiently early (ie, >30 days before delivery).^6^ As a public health intervention, antenatal syphilis screening and treatment is cost-saving in moderate and high prevalence settings, and cost-effective even when prevalence is less than 1%.^7–10^

Unfortunately, congenital syphilis is much harder to diagnose than syphilis in pregnant women, and therefore estimates are needed to accurately reflect burden of disease in most countries. Global maternal and congenital syphilis estimates were published in 2007 using peer-reviewed and unpublished country reports available from 31 countries from 1997 to 2003 in which studies used serologically confirmed tests in antenatal care attendees. The 2007 estimates suggested that more than 2 million maternal and 1–5 million congenital syphilis cases would occur every year worldwide without treatment.^11^ These estimates served as the basis for the 2007 launch of a global initiative for the elimination of mother-to-child transmission of syphilis and congenital syphilis, which has resulted in worldwide policy and programme changes.

In 2008, the WHO and global partners introduced surveillance of syphilis in pregnancy within the HIV Universal Access and Global AIDS Response Progress Reporting (UA/GARPR) system to monitor progress. Countries were encouraged to report data for three core indicators collected on antenatal care attendees: syphilis testing, seropositivity, and treatment (added in 2010).^12^

To measure progress for the new initiative, the 2008 baseline global and regional estimates of syphilis in pregnancy used antenatal syphilis seropositivity data reported by 96 countries to the UA/GARPR. These 2008 estimates noted 1 4 million maternal and 520 000 congenital syphilis infections, including 304000 perinatal deaths, occurred globally.^13^ The 2008 estimates uniformly adjusted for seropositivity to minimise overdiagnosis of active syphilis. Furthermore, the 2008 estimates used fixed regional assumptions for testing and treatment coverage.

We aimed to estimate the global and regional cases and prevalence of maternal and congenital syphilis in 2012. With stronger surveillance and better country-level reporting over time, we also used the same method to revise the 2008 (baseline) estimates, and compared these updated 2008 estimates with the new 2012 estimates to assess progress made in the global elimination initiative.

## Methods

### Data inputs

In this analysis we used a health systems model approved by the Child Health Epidemiology Reference Group in June, 2011, and by technical experts at the WHO meeting on sexually transmitted infection estimates held in November, 2013. Whenever possible, we used actual country data reported by the 194 WHO member states and where data were not available, we imputed numbers based on regional estimates (appendix); where no data were available for southern Latin America, we used the regional mean for tropical Latin America; where no data were available for high-income North America, Australasia, or high-income Asia Pacific, we used the regional mean for western Europe; where no data were available for east Asia, we used the regional mean for southeast Asia. For each country, we obtained the numbers of livebirths from the United Nations Development Programme World Population Prospects database.^14^ Cousens and colleagues^15^ provided the estimated national numbers of stillbirths (defined as fetal death in the third trimester, with fetal weight at least 1000 g or gestation at least 28 completed weeks).

We obtained antenatal clinic attendance (at least one visit) from the WHO Global Health Observatory.^16^ We used most recently collected UA/GARPR data from 2000–08 for updated 2008 and 2009–12 for 2012 estimates. The UA/GARPR provided country data for three variables: syphilis seropositivity with specified test type (non-treponemal, treponemal, or both), testing of antenatal care attendees for syphilis, and treatment of syphilis seropositive antenatal care attendees. When seropositivity data were unavailable, we searched PubMed for peer-reviewed reports and the grey literature between Jan 1, 2000, and March 18, 2014, using the search terms (“syphilis” OR “treponemal” OR “non-treponemal” AND “[country name]” and added [“pregnant” OR “perinatal” OR “antenatal”]. This search provided seropositivity data for eight countries.^17–24^ For updated 2008 estimates, we used the testing data with the treatment assumption used for the original 2008 estimates.^13^ When data were unavailable for these variables, we used the most recent country values to calculate regional means for 21 Institute for Health Metrics and Evaluation (IHME) regions for imputation. IHME regions categorise countries based on epidemiological similarity and geographical proximity.^25^ However, we presented these data for the six WHO regional groupings to facilitate WHO regional programmatic and planning processes.

The appendix shows the model input values and regional means by WHO and IHME regions used to generate the updated 2008 and 2012 estimates. We used the following assumptions based on published scientific literature to generate estimates: first, we defined probable active syphilis infections as cases reactive on both treponemal and non-treponemal serological tests. We applied separate correction factors for four different scenarios by the country-specified test type to adjust for possible error due to the diagnostic test type used: both reactive non-treponemal and reactive tests (1 0); only reactive non-treponemal (0 522); only reactive treponemal tests (0 536); and neither test reported (0 686; appendix). This correction factor was based on a random effects modelling analysis of positivity data obtained through a recent review of the scientific literature.^26^

Second, the number of early fetal deaths (defined as occurring at 22 through 28 weeks’ gestation) was estimated at 20% of stillbirths based on the relationship of early to late fetal deaths recorded in multicountry reviews (mainly high-income countries).^15,27,28^ We estimated the number of pregnancies by summing livebirths, stillbirths, and early fetal deaths.

Third, the expected proportions of pregnancies resulting in any adverse pregnancy outcomes (52%) included early fetal deaths or stillbirths (21%), preterm (born alive before 37 weeks’ gestation) or low birthweights (weighing <2500 g; 6%), neonatal death (death in first 28 days; 9%, or clinical disease in infants (29–365 days; 16%, of syphilis-infected women and were based on a recent meta-analysis.^3^

Finally, the effectiveness of penicillin at preventing any adverse pregnancy outcome (84%, the weighted average using the expected proportions as the weights) included early fetal deaths or stillbirths (82%), preterm or low birthweights (64%;, neonatal deaths (80%, and clinical disease in infants (97%, and was based on a published literature review.^6^

In sum, this method for updated 2008 and 2012 estimates was identical to the health systems model used for the original WHO 2008 estimates except that it used newly available, country-reported testing data and country-reported treatment data (2012 only); a more nuanced, evidence-based correction factor for test type; and IHME regional classifications for imputing means rather than medians for countries without data.^13^

### Analyses

We used a four-step process to estimate for 2012, and update for 2008, the number of pregnant women with probable active syphilis infections and associated adverse pregnancy outcomes for each country. We summed country values to N, where N equals the number of countries for each of the six WHO regions and 194 WHO member countries for the global total.

For step 1 we estimated the number of pregnant women with probable active syphilis infections.

$$\sum_{c=1}^{N}PASI_{c},where\;PASI_{c}=\left(Pos_{c}\right)\left(cf_{c}\right)\left(preg_{c}\right)$$

In step 2 we estimated the number of pregnant women with probable active syphilis infections attending antenatal care;

$$\sum_{c=1}^{N}PASI_{-}ANC_{c},where\;PASI_{-}ANC_{c}=\left(PASI_{c}\right)\left(ANC1_{c}\right)$$

among antenatal care attendees, we estimated the numbers of pregnant women with syphilis infections who were not tested; tested but not treated; and tested and treated for syphilis:

$$\begin{array}{l} \sum_{c=1}^{N}PASI_{-}ANC_{-}NT_{c},where\;PASI_{-}ANC_{-}NT_{c}=\left(PASI_{-}ANC_{c}\right)\left(1-Tested_{c}\right)\\ \sum_{c=1}^{N}PASI_{-}ANC_{-}{\text{TNT}}_{c},where\;PASI_{-}ANC_{-}TNT_{c}=\left(PASI_{-}ANC_{c}\right)\left(\;Tested{\;}_{c}\right)\left(\;1-Treated{\;}_{c}\right)\\ \sum_{c=1}^{N}PASI_{-}ANC_{-}TT_{c},where\;PASI_{-}ANCTT_{c}=\left(PASI_{-}ANC_{c}\right)\left(\;Tested{\;}_{c}\right)\left(\;Treated{\;}_{c}\right)\\ \sum_{c=1}^{N}PASI_{-}ANC_{-}TT_{c},\;where\;{PASI}_{-}{ANC}_{-}TT_{c}=(PASI_{-}ANC_{c})\;(Tested_{c})(Treated_{c})\\ \sum_{c=1}^{N}PASI_{-}ANC_{-}TNT_{c},where\;PASI_{-}ANC_{-}TNT_{c}=\left(PASI_{-}ANC_{c}\right)\left(Teted_{c}\right)\left(1-Treated_{c}\right) \end{array}$$

And for pregnant women with syphilis who did not access antenatal care services.

$$\sum_{c=1}^{N}PASI_{-}NOANC_{c},where\;PASI_{-}NOANC=\left(PASI_{c}\right)\left(1-ANC1_{c}\right)$$

In step 3, we estimated the numbers of adverse pregnancy outcomes for syphilis-infected pregnant women attending antenatal care who were not tested; tested but not treated; and tested and treated but in whom treatment was ineffective at preventing an adverse pregnancy outcome:

$$\sum_{c=1}^{N}AO_{-}ANC_{-}NT_{c},where\;AO_{-}ANC_{-}NT_{c}=\left(PASI_{-}ANC_{-}NT_{c}\right)\left(P_{EFD,SB}+P_{P,LBW}+P_{NND}+P_{I}\right)\;\sum_{c=1}^{N}AO_{-}ANC_{-}TNT_{c},where\;AO_{-}ANC_{-}TNT_{c}=\left(PASI_{-}ANC_{-}TNT_{c}\right)\left(P_{EFDSB}+P_{P,LBW}+P_{NND}+P_{I}\right)\;\sum_{c=1}^{N}AO_{-}ANC_{-}TT_{c},where\;AO_{-}ANC_{-}TT_{c}=\left(PASI_{-}ANC_{-}TT_{c}\right)(\left(P_{EFD,SB}\right)(\left(\left(P_{EFD,SB}\right)\left(1-E_{EFD,SB}\right)\right)+\left(\left(P_{P,LBW}\right)\left(1-E_{P,LBW}\right)\right)+\left(1-E_{NND}\right))+\left(\left(P_{I}\right)\left(1-E_{I}\right)\right))$$

And for pregnant women with syphilis who did not access antenatal care services:

$$\sum_{c=1}^{N}AO_{-}NOANC_{c},where\;AO_{-}NOANC_{c}=\left(PASI_{-}NOANC_{c}\right)\left(P_{EFD,SB}+P_{P,LBW}+P_{NND}+P_{I}\right)$$

For step 4, for the total number of adverse outcomes associated with syphilis in pregnancy, we summed adverse outcomes among women who did not attend antenatal care and antenatal care attendees who were not tested, tested but not treated, and tested and treated for syphilis:

$$\sum_{c=1}^{N}AO,where\;AO_{c}=AO_{-}NOANC_{c}+AO_{-}ANC_{-}NT_{c}+AO_{-}ANC_{-}TNT_{c}+AO_{-}ANC_{-}TT_{c}$$

To calculate maternal and congenital syphilis prevalence, we divided the respective case estimates by the estimated number of pregnancies.

For analytical purposes, we calculated the percent change between updated 2008 and 2012 estimates worldwide, for six WHO regions, and 194 countries to examine which countries were driving the global change.

To assess robustness of trends in the number of pregnant women with active syphilis infections from the updated 2008 to 2012 estimates, we did two additional analyses: all countries, irrespective of whether data were reported values or regional means (to compare the overall differences between estimates); and only countries with seropositivity values in both updated 2008 and 2012 estimates (to assess trends in consistently reporting countries).

We did the same two analyses for adverse pregnancy outcomes for countries reporting seropositivity and testing data in both time periods and also included countries with reported treatment data in 2012. To assess the validity of the treatment assumption used in updated 2008 estimates, we recalculated proportions using the 2012 treatment data. To describe the burden of syphilis based on the global distribution of wealth, we stratified countries by World Bank income groups.^29^

We did not perform a formal uncertainty analysis as there were insufficient data beyond those used in this analysis to allow for evidence-based calculation of uncertainty.

### Role of the funding source

The funders had no role in the study design, conduct, analysis, or writing ofthe manuscript. The corresponding author had full access to the data and had final responsibility for the decision to submit for publication.

## Results

**Figure 1:**
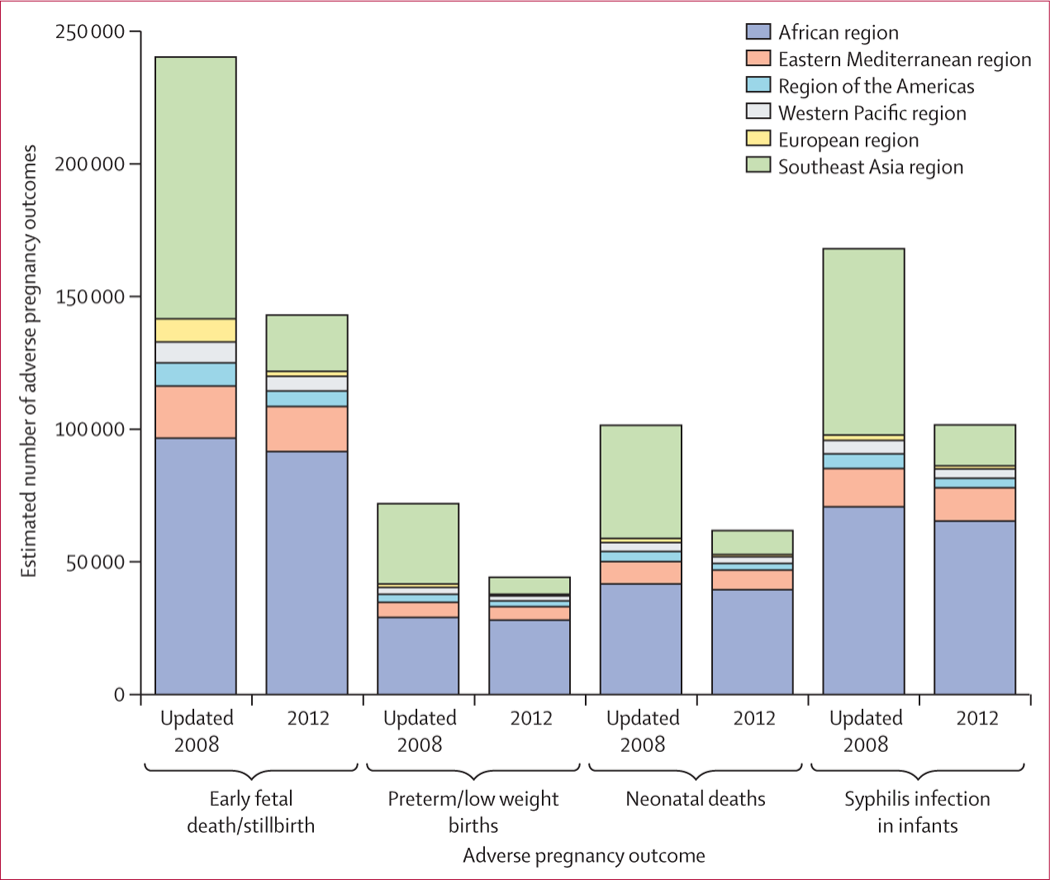
Estimated numbers of syphilis-associated adverse pregnancy outcomes by WHO region in 2008 and 2012

From 2008 to 2012, the number of reporting countries increased from 88 to 122 for syphilis seropositivity, nine to 47 for test type, 58 to 106 for testing, and zero (not reported until 2010) to 74 countries for treatment. Data availability increased in all WHO regions. In 2012, we estimated that 927936 pregnant women (0 66% prevalence) had probable active syphilis infections, resulting in 350 915 adverse pregnancy outcomes. These outcomes included 143 100 early fetal deaths, 44 132 preterm or low birthweight infants, 61 870 neonatal deaths, and 101 813 infected infants (figure 1; for the full country dataset see appendix).

**Figure 2:**
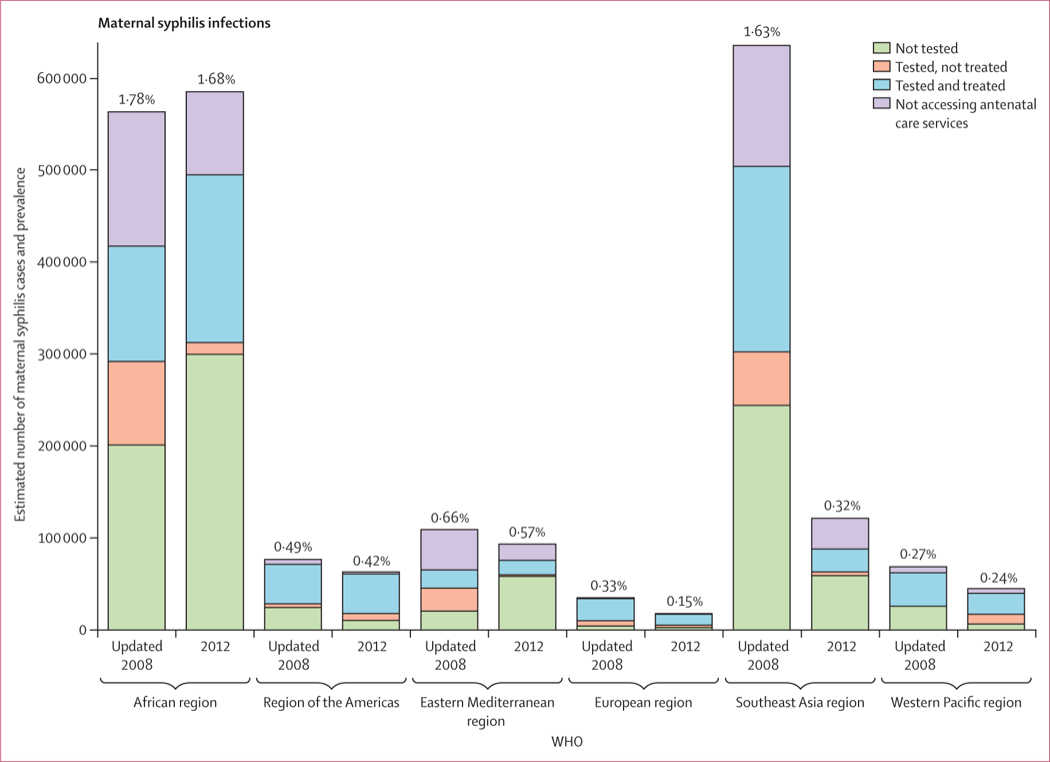
Estimated number of maternal syphilis cases and prevalence by WHO region

**Figure 3:**
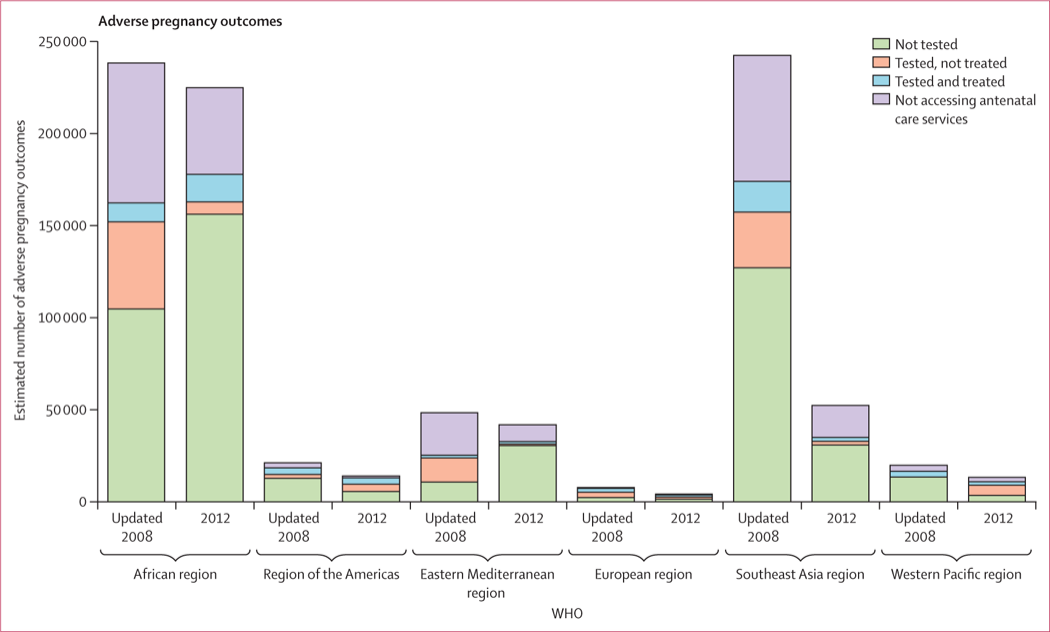
Estimated number of syphilis-associated adverse pregnancy outcome by WHO region

The distribution of maternal syphilis infections and adverse outcomes varied across regions (figures 2 and 3). The burden of syphilis in pregnancy was greatest in Africa, representing 63 1% of global maternal infections (585 664; 1 68% prevalence) and 64 0% of adverse outcomes (224761; 0 66%), and was lowest in Europe, representing 2 0% of maternal infections (18437; 0 15%) and 1 2% of adverse outcomes (4307; 0 04%).

Worldwide in 2012, an estimated 84 6% of pregnant women with syphilis (779 079 of 927 936 women) attended antenatal care at least once, of whom 56 4% were not tested for the infection (439 412 of 779 079); these cases resulted in 65 1% (228 494) of all 350 915 adverse outcomes. 5 0% (39 046 of 779 079) of antenatal care attendees with syphilis were tested but not treated, resulting in 5 8% (20 304 infants) of all 350 915 adverse outcomes. Additionally, 38 6% (300 621 of 779 079) of pregnant women with syphilis who were tested and treated in antenatal care, but for whom treatment was ineffective (eg, given too late in pregnancy to prevent an adverse outcome, or given oral treatment rather than sufficient intramuscular doses of benzathine benzylpenicillin) contributed to 7 0% of all adverse outcomes (24711). The 16 0% (148 857 of 927 936) of pregnant women with syphilis who did not access antenatal care accounted for 22 1% (77 406 of 350 915) of adverse outcomes. Overall, 96 6% (896 630 of 927 936) of maternal syphilis infections and 98 1% (344 474 of 350 916) of adverse outcomes occurred in low-income and middle-income countries.

**Figure 4:**
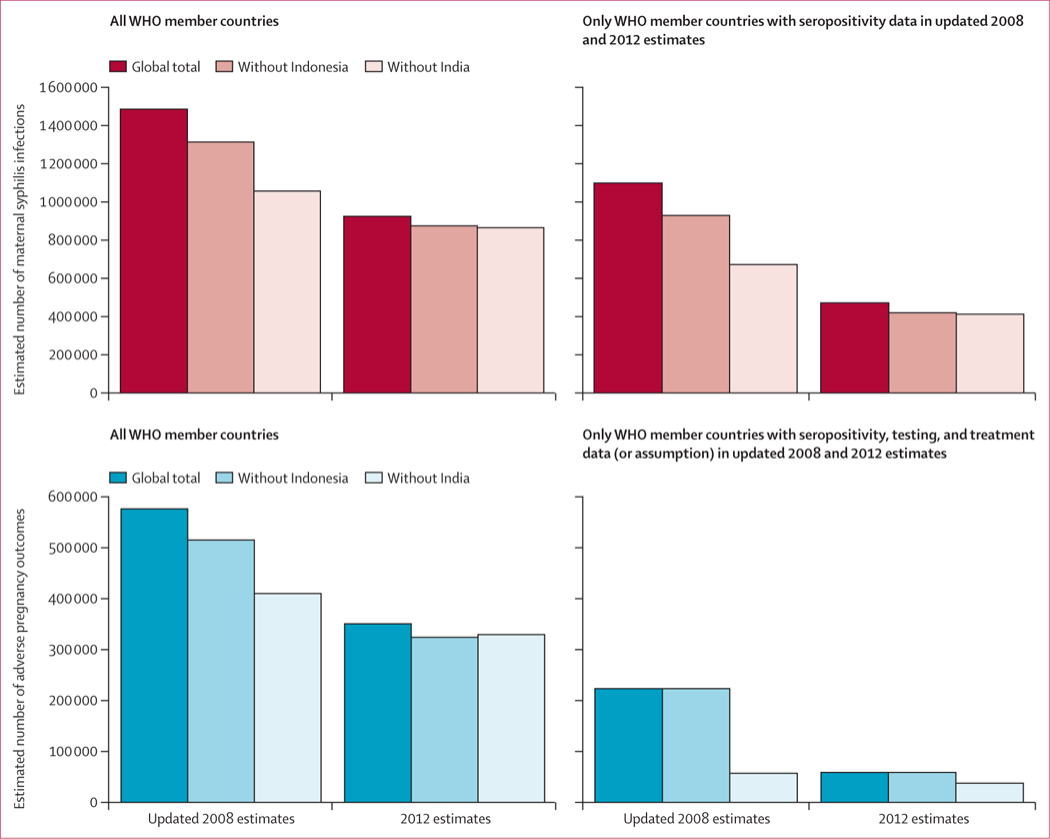
Estimated number of maternal syphilis (A) and any associated adverse pregnancy outcome (B) for all countries and only countries with data

From updated 2008 to 2012 estimates, maternal syphilis infections and adverse outcomes decreased for all scenarios studied (figure 4). Using data for all WHO member countries, from updated 2008 to 2012 estimates, maternal infections decreased by 37 7% (from 1488 394 [1 07% prevalence] in 2008 to 927 936 [0 66%] in 2012) and adverse outcomes decreased by 39 2% (576 784 in 2008 to 350 915 in 2012). Decreases were notable in southeast Asia (80 9% decrease in maternal infections; 78 4% decrease in adverse outcomes; figures 2 and 3). The proportion of pregnant women not tested for syphilis in antenatal care fell in all regions except Africa (which increased by 49 0% from 201 547 to 300 246). When we considered only the 73 countries reporting data for both 2008 and 2012, we noted a 57 1% decrease in maternal infections (1102 261 to 473 030).

We examined data from the ten countries with the greatest numbers of livebirths to assess their effect on global and regional estimates (appendix). From updated 2008 to 2012 estimates, the change in seropositivity of antenatal care attendees in India (from 2 3% to 0 4%) and Indonesia (from 5 8% to 1 2%) resulted in 489 621 fewer maternal infections and 180 107 fewer adverse outcomes. India alone accounted for 65 8% of the global difference in maternal infections and 64 4% of adverse outcomes between updated 2008 and 2012 estimates.

Excluding India in reanalysis in updated 2008 and 2012 estimates resulted in a decrease in maternal infections by 18 1% (from 1059 327 to 867 924 cases) and in adverse outcomes by 17 8% (401 098 to 329 683 cases; figure 4). Countries with data available for both updated 2008 and 2012 estimates had a 38 6% decrease in maternal infections (from 673 194 to 413 018) and 34 7% decrease in adverse outcomes (from 56 781 to 37 094). Even if we assumed the higher treatment values reported in 2012 had also occurred in 2008, adverse outcomes still fell by more than 20%.

## Discussion

This analysis suggests progress in eliminating mother-to-child transmission of syphilis, with more than one-third fewer maternal and congenital syphilis infections from 2008 to 2012. Since the launch of the global initiative, more countries have introduced universal syphilis screening in pregnancy, often using rapid syphilis testing algorithms when laboratory capacity is limited. More countries have integrated prevention of mother-to-child transmission of HIV and syphilis into maternal health programmes. Additionally, surveillance of antenatal syphilis has improved greatly. India and Indonesia represented a large proportion of the global decreases; however, decreased surveillance persisted even when data for India were excluded.

Despite this positive momentum, syphilis continues to affect large numbers of pregnant women and result in substantial perinatal morbidity and mortality. In 2012, nearly 1 million pregnant women had syphilis infections and roughly 350 000 had a syphilis-induced adverse pregnancy outcome despite incremental improvements in screening and treatment. Most syphilis-induced pregnancy outcomes were in women who attended antenatal care, suggesting that these poor outcomes could have been prevented had recommended testing and treatment been done. Women and infants in low-income and middle-income countries suffered disproportionately, and progress in Africa was especially slow.

Our data suggested improvement in coverage of antenatal syphilis screening and treatment from 2008 to 2012 in most regions worldwide. Nonetheless, many countries were far from achieving the global programme targets (at least 95% screening in pregnant women and 95% treatment in women testing positive).^30,31^ Although more than three-quarters of pregnant women with syphilis in 2012 had at least one antenatal care visit, more than half of these women either did not receive syphilis testing or received inadequate treatment to prevent congenital syphilis. These systemic inadequacies in antenatal care led to nearly 80% of global congenital syphilis cases (nearly 275 000 events). This shortcoming is a failure of the public health system and a clarion call for improving the quality of antenatal care services.

Global monitoring is improving, with many more countries reporting data for each of the three core indicators in 2012 than in 2008. However, 41% of countries had not reported syphilis seropositivity data, 45% had not reported testing coverage, and 62% had not reported treatment coverage through the UA/GARPR system. Increased efforts are needed to strengthen surveillance and monitoring for the elimination of mother-to-child transmission of syphilis.^12,32,33^

**Figure 5:**
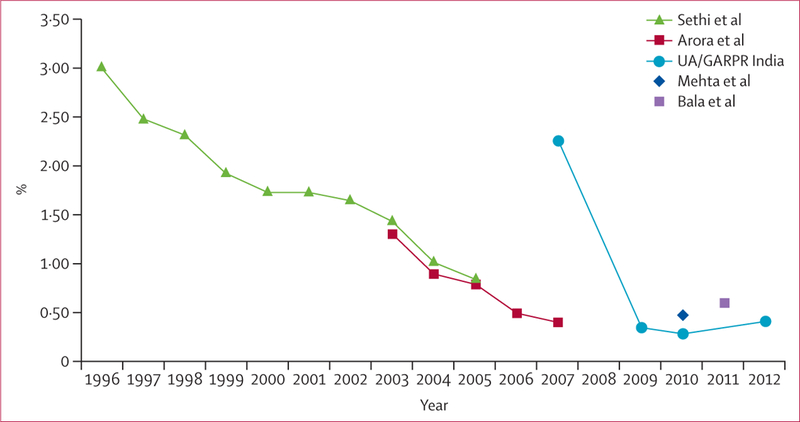
Prevalence of syphilis in pregnant women in India from various sources, 1996–2012^33–36^ UA/GARPR=Universal Access and Global AIDS Response Progress Reporting.

Our analysis has limitations. First, the syphilis seropositivity datapoint reported by India and used in the updated 2008 estimates might have been spuriously high. Three consecutive lower values in UA/GARPR and a scientific literature review from India on syphilis prevalence in pregnancy supports this possibility (figure 5).^33–36^ Two studies in India suggest decreases in syphilis in pregnancy occurred before 2007. Sethi and co-authors^33^ noted that the prevalence of syphilis in pregnancy in a tertiary health-care centre in northern India decreased from 3 0% in 1996 to 0 84% in 2005. They attributed the decrease to educating women on the risks of syphilis in antenatal care, monitoring management of sexually transmitted infections, and more widespread use of over-the-counter antibiotics. In another study, Arora and colleagues^34^ suggested that targeted interventions for female sex workers implemented by the National AIDS Control Organization and the Avahan Project beginning in 1995 had reduced the prevalence of syphilis in young pregnant women from 1 3% in 2003 to 0 4% in 2007. Additionally, although no peer-reviewed scientific literature on Indonesia reported syphilis seropositivity in antenatal care attendees, the metadata for the UA/GARPR datapoints suggested that syphilis surveillance in antenatal care settings has expanded since 2008. The 2008 data were from a pilot project to increase screening for HIV and syphilis in antenatal care in three provinces, whereas the 2009 data represented an expanded pilot in five provinces. Selected for high HIV prevalence, these provinces might have a higher prevalence of syphilis than the national average. Such challenges in obtaining accurate and nationally representative data are unlikely to be unique to India and Indonesia.

Second, although completeness of reporting of syphilis seropositivity in antenatal care attendees through the UA/GARPR system improved from 2008 to 2012, two populous countries—Pakistan and the USA—did not report to WHO during this period. Pakistan does not currently have a policy of universal syphilis screening in antenatal care, so nationally representative, routinely collected data are unavailable. One local study of syphilis seropositivity in antenatal care attendees in Karachi, Pakistan, reported 0 9%, which is substantially greater than the regional mean (0 2%) for the eastern Mediterranean region.^37^ The USA did not report antenatal syphilis data to UA/GARPR, but the US national case reporting system in 2012 recorded 659 fewer congenital syphilis cases than our 2012 estimates of adverse pregnancy outcomes.^38^ This discrepancy is not substantial enough to have had an important effect on global or regional estimates.

Third, the test type used to define positivity was unavailable for one-quarter of syphilis seropositivity values. Although we adjusted for test type to account for this, this paucity of data increases the uncertainty of our estimates. Similar data quality issues likely exist for other datapoints underlying this modelling exercise, and might affect our estimates. We hope that future rounds of estimates will be able to better explore ways to capture this uncertainty.

Finally, interpretation of trends in treatment coverage is difficult because we did not have country-reported data for 2008, and relied on regional treatment assumptions. For example, in Africa, although we are confident that screening decreased regionally from 2008 to 2012, we cannot be certain that treatment of maternal syphilis worsened over time.

In summary, these new global estimates of maternal and congenital syphilis suggest decreases from 2008 to 2012 worldwide and across most WHO regions. However, our findings also suggest that untreated maternal syphilis remains a substantial cause of preventable perinatal morbidity and mortality. Progress was especially limited in Africa, the region with the greatest congenital syphilis burden. Improving access to and the quality of early antenatal care, increasing syphilis testing at first antenatal care visit, ensuring adequate and prompt treatment for infected women and their partners, and expanding programmes of targeted interventions for high-risk groups are necessary to improve control of the elimination of mother-to-child transmission of syphilis. Additionally, nationally representative data on the three core indicators are important for understanding global, regional, and country-level progress in eliminating this old, but still important, public health problem.

## Supplementary Material

Supplemental Appendix 2

## Research in context

### Evidence before this study

In 2007, WHO launched the global Elimination of Congenital Syphilis initiative, with a goal of reducing mother-to-child transmission of syphilis to a sufficiently low level that congenital syphilis was no longer a public health problem. The goal is for countries to achieve a congenital syphilis rate of 50 or fewer cases per 100 000 livebirths through achieving programmatic targets of more than 95% syphilis screening coverage in pregnancy and more than 95% treatment of seropositive women. In 2008, surveillance of syphilis in pregnancy was included within the HIV Universal Access and Global AIDS Response Progress Reporting (UA/GARPR) system. Countries were encouraged to report data for three core indicators collected on antenatal care attendees: syphilis testing, seropositivity, and treatment. Baseline global and regional estimates of syphilis in pregnancy for 2008, which used antenatal syphilis seropositivity data reported by 96 countries to the UA/GARPR, found 1–4 million maternal and 520 000 congenital syphilis infections globally, including 304 000 perinatal deaths. In this analysis, we used the most recently collected GARPR data (2009–12) for key congenital syphilis indicators for 2012 estimates, and compared these against 2008 updated baseline data from GARPR (2000–08), the start of the elimination effort.

### Added value of this study

The findings of our current analysis add value to the existing body of evidence by providing a 5-year, interim report on progress made with the launch of the global initiative for the elimination of congenital syphilis. The analysis found a 38% reduction in the number of pregnant women with syphilis and in the number of congenital syphilis cases. Global monitoring improved from 2008 to 2012, with more countries reporting on sexually transmitted infection indicators. Nonetheless, certain countries experienced challenges and may need support. Updated estimates of the baseline global burden of maternal and congenital syphilis (2008) using GARPR data were available for more countries. The study presents an acceptable model vetted by experts for estimating progress on the global elimination of congenital syphilis initiative for use in future updates.

### Implications of all the available evidence

These results help policy makers and leaders understand where challenges remain and can be addressed. They can be used for broader global maternal and child health programmes, in which the extent of congenital syphilis (still accounting for almost 200 000 perinatal deaths each year) is often overlooked. Regions and countries can use our data to advocate for the needed resources to carry out routine screening of syphilis in antenatal care in all countries, including some newer screening applications such as dual rapid HIV and syphilis tests on a single device, and to estimate treatment needs.

## References

[1] Fiumara NJ. Syphilis among mothers and children. Ann N Y Acad Sci 1988; 549: 187–92. 3228253 10.1111/j.1749-6632.1988.tb23971.x

[2] Chakraborty R, Luck S. Syphilis is on the increase: the implications for child health. Arch Dis Child 2008; 93: 105–09. 18208988 10.1136/adc.2006.103515

[3] Gomez GB, Kamb ML, Newman LM, Mark J, Broutet N, Hawkes SJ. Untreated maternal syphilis and adverse outcomes of pregnancy: a systematic review and meta-analysis. Bull World Health Organ 2013; 91: 217–26. 23476094 10.2471/BLT.12.107623 PMC3590617

[4] Woods CR. Congenital syphilis-persisting pestilence. Pediatr Infect Dis J 2009; 28: 536–37 19483520 10.1097/INF.0b013e3181ac8a69

[5] Zenker PN, Rolfs RT. Treatment of syphilis, 1989. Rev Infect Dis 1990; 12 (suppl 6): S590–609. 2201075 10.1093/clinids/12.supplement_6.s590

[6] Blencowe H, Cousens S, Kamb M, Berman S, Lawn JE. Lives Saved Tool supplement detection and treatment of syphilis in pregnancy to reduce syphilis related stillbirths and neonatal mortality. BMC Public Health 2011; 11 (suppl 3): S9. 10.1186/1471-2458-11-S3-S9 PMC3231915 21501460

[7] WHO. The Global Elimination of Congenital syphilis: rationale and strategy for action. Geneva: World Health Organization, 2007.

[8] Schackman BR, Neukermans CP, Fontain SN, Cost-effectiveness of rapid syphilis screening in prenatal HIV testing programs in Haiti. PLoS Med 2007; 4: e183. 17535105 10.1371/journal.pmed.0040183 PMC1880854

[9] World Bank. World development report 1993: Investing in health. New York: World Bank, 1993.

[10] WHO. Investment case for eliminating mother-to-child transmission of syphilis: Promoting better maternal and child health and stronger health systems. Geneva: World Health Organization, 2012.

[11] Schmid GP, Stoner BP, Hawkes S, Broutet N. The need and plan for global elimination of congenital syphilis. Sex Transm Dis 2007; 34 (suppl): S5–10. 17592390 10.1097/01.olq.0000261456.09797.1b

[12] WHO. Methods for surveillance and monitoring of congenital syphilis elimination within existing systems. Geneva: World Health Organization, 2011.

[13] Newman L, Kamb M, Hawkes S, Global estimates of syphilis in pregnancy and associated adverse outcomes: analysis of multinational antenatal surveillance data. PLoS Med 2013; 10: e1001396. 10.1371/journal.pmed.1001396 PMC3582608 23468598

[14] United Nations. The World Population Prospects -the 2010 revision. http://unstats.un.org/unsd/mdg/Resources/Static/Data/MDGRegionCodes_200611.xls (accessed June 28, 2011).

[15] Cousens S, Blencowe H, Stanton C, National, regional, and worldwide estimates of stillbirth rates in 2009 with trends since 1995: a systematic analysis. Lancet 2011; 377: 1319–30. 21496917 10.1016/S0140-6736(10)62310-0

[16] WHO. Antenatal care coverage: at least one visit (%). http://apps.who.int/gho/data/node.main.492?lang=en (accessed Oct 16, 2013).

[17] Kuznik A, Lamorde M, Nyabigambo A, Manabe YC. Antenatal syphilis screening using point-of-care testing in Sub-Saharan African countries: a cost-effectiveness analysis. PLoS Med 2013; 10: e1001545. 10.1371/journal.pmed.1001545 PMC3818163 24223524

[18] Republic of Senegal National Council for the Fight Against AIDS Ministry of Health and Medical Prevention Division of the Fight Against AIDS and STIs. 2009 Sentinel surveillance of HIV and syphilis in pregnant women in Senegal. Epidemiological Bulletin No. 14; Dakar, Senegal.

[19] Strasser S, Bitarakwate E, Gill M, Introduction of rapid syphilis testing within prevention of mother-to-child transmission of HIV programs in Uganda and Zambia: a field acceptability and feasibility study. J Acquir Immune Defic Syndr 2012; 61: e40–6. 22820810 10.1097/QAI.0b013e318267bc94

[20] Caddy SC, Lee BE, Sutherland K, Pregnancy and neonatal outcomes of women with reactive syphilis serology in Alberta, 2002 to 2006. J Obstet Gynaecol Can 2011; 33: 453–59. 21639965 10.1016/S1701-2163(16)34878-2

[21] Sampedro A, Mazuelas P, Rodriguez-Granger J, Torres E, Puertas A, Navarro JM. Serological markers in immigrant and Spanish pregnant women in Granada. Enferm Infecc Microbiol Clin 2010; 28: 694–97 (in Spanish). 20961669 10.1016/j.eimc.2010.04.007

[22] Kwan KS, Giele CM, Combs B, Mak DB. Improvement in antenatal testing for sexually transmissible infections and blood-borne viruses in Western Australian hospitals, 2007 to 2010. Sex Health 2012; 9: 349–54. 22877594 10.1071/SH11151

[23] Cho YH, Kim HO, Lee JB, Lee MG. Syphilis prevalence has rapidly decreased in South Korea. Sex Transm Infect 2003; 79: 323–24. 12902586 10.1136/sti.79.4.323 PMC1744720

[24] Cliffe SJ, Tabrizi S, Sullivan EA, and the Pacific Islands Second Generation HIV Surveillance Group. Chlamydia in the Pacific region, the silent epidemic. Sex Transm Dis 2008; 35: 801–06. 18580823 10.1097/OLQ.0b013e318175d885

[25] Murray CJ, Ezzati M, Flaxman AD, GBD 2010: design, definitions, and metrics. Lancet 2012; 380: 2063–66. 23245602 10.1016/S0140-6736(12)61899-6

[26] Ham DC, Lin C, Newman L, Wijesooriya NS, Kamb M. Improving global estimates of syphilis in pregnancy by diagnostic test type: A systematic review and meta-analysis. Int J Gynaecol Obstet 2015; 130 (suppl 1): S10–14. 25963909 10.1016/j.ijgo.2015.04.012 PMC4591031

[27] Flenady V, Koopmans L, Middleton P, Major risk factors for stillbirth in high-income countries: a systematic review and meta-analysis. Lancet 2011; 377: 1331–40. 21496916 10.1016/S0140-6736(10)62233-7

[28] Lawn JE, Blencowe H, Pattinson R, , and The Lancets Stillbirths Series steering committee. Stillbirths: Where? When? Why? How to make the data count? Lancet 2011; 377: 1448–63. 21496911 10.1016/S0140-6736(10)62187-3

[29] World Bank. Country and Lending Groups. http://data.worldbank.org/about/country-and-lending-groups (accessed May 14, 2014).

[30] WHO. Global guidance on criteria and processes for validation: elimination of mother-to-child transmission (the elimination of mother-to-child transmission) of HIV and syphilis. Geneva: World Health Organization, 2014.

[31] Joint United Nations Programme on HIV/AIDS. Global AIDS response progress reporting 2014: construction of core indicators for monitoring the 2011 UN political declaration of HIV /AIDS. Geneva: Joint United Nations Programme on HIV/AIDS, 2014.

[32] Hawkes S, Matin N, Broutet N, Low N. Effectiveness of interventions to improve screening for syphilis in pregnancy: a systematic review and meta-analysis. Lancet Infect Dis 2011; 11: 684–91. 21683653 10.1016/S1473-3099(11)70104-9

[33] Sethi S, Sharma K, Dhaliwal LK, Banga SS, Sharma M. Declining trends in syphilis prevalence among antenatal women in northern India: a 10-year analysis from a tertiary healthcare centre. Sex Transm Infect 2007; 83: 592. 10.1136/sti.2007.025551 PMC2598655 18024713

[34] Arora P, Nagelkerke NJ, Moineddin R, Bhattacharya M, Jha P. Female sex work interventions and changes in HIV and syphilis infection risks from 2003 to 2008 in India: a repeated cross-sectional study. BMJ Open 2013; 3: DOI:10.1136/bmjopen-2013-002724 PMC3686231 23794571

[35] Mehta KD, Antala S, Mistry M, Goswami Y. Seropositivity of hepatitis B, hepatitis C, syphilis, and HIV in antenatal women in India. J Infect Dev Ctries 2013; 7: 832–37 24240041 10.3855/jidc.2764

[36] Bala M, Singh V, Muralidhar S, Ramesh V. Assessment of reactivity of three treponemal tests in non-treponemal non-reactive cases from sexually transmitted diseases clinic, antenatal clinic, integrated counselling and testing centre, other different outdoor patient departments/indoor patients of a tertiary care centre and peripheral health clinic attendees. Indian J Med Microbiol 2013; 31: 275–79. 23883715 10.4103/0255-0857.115638

[37] Shah SA, Kristensen S, Memon MA, Prevalence of syphilis among antenatal clinic attendees in Karachi: imperative to begin universal screening in Pakistan. J Pak Med Assoc 2011; 61: 993–97. 22356034 PMC3574871

[38] US Centers for Disease Control and Prevention. Sexually Transmitted Disease Surveillance 2012. Atlanta: Centers for Disease Control and Prevention, 2013.

